# Glyceraldehyde-3-phosphate dehydrogenase/1,3-bisphosphoglycerate-NADH as key determinants in controlling human retinal endothelial cellular functions: Insights from glycolytic screening

**DOI:** 10.1016/j.jbc.2025.108472

**Published:** 2025-03-28

**Authors:** Nicole Oska, Ahmed M. Awad, Shaimaa Eltanani, Mohamed Shawky, Armaan Naghdi, Thangal Yumnamcha, Lalit Pukhrambam Singh, Ahmed S. Ibrahim

**Affiliations:** 1 Department of Ophthalmology, Visual, and Anatomical Sciences, School of Medicine, Wayne State University, Detroit, Michigan, USA; 2 Department of Pharmacology and Toxicology, Faculty of Pharmacy, Mansoura University, Mansoura, Egypt; 3 Department of Pharmacology and Toxicology, Faculty of Pharmacy, Mansoura National University, Gamasa, Egypt; 4 Department of Biochemistry, Faculty of Pharmacy, Horus University, New Damietta, Egypt; 5 Department of Biochemistry, Faculty of Pharmacy, Mansoura University, Mansoura, Egypt; 6 Department of Pharmacology, School of Medicine, Wayne State University, Detroit, Michigan, USA; 7 Molecular Therapeutics Research Program, Karmanos Cancer Institute (KCI), School of Medicine, Wayne State University, Detroit, Michigan, USA

**Keywords:** AZD3965, barrier integrity, cell spreading, diabetic retinopathy (DR), electric cell-substrate impedance sensing (ECIS), endothelial cell, galloflavin, glycolysis, heptelidic acid, MSDC-0602, NG52, pyruvate kinase, retina, shikonin

## Abstract

Maintaining barrier integrity, along with cell adhesion to the extracellular matrix and the subsequent process of cell spreading, are essential functions of endothelial cells, including human retinal endothelial cells (HRECs). Disruptions in these processes can lead to vision-threatening conditions like diabetic retinopathy. However, the bioenergetic mechanisms that regulate HREC barrier function and cell spreading remain incompletely understood. This study investigates the role of lower glycolytic components in modulating these critical functions of HRECs. *In vitro*, Electric Cell-Substrate Impedance Sensing (ECIS) technology was used to measure real-time changes in HREC barrier integrity (electrical resistance) and cell spreading (capacitance). Pharmacological inhibitors targeting lower glycolytic components were tested: heptelidic acid for glyceraldehyde-3-phosphate dehydrogenase (GAPDH), NG-52 for phosphoglycerate kinase (PGK), shikonin for pyruvate kinase M (PKM), galloflavin for lactate dehydrogenase (LDH), AZD3965 for lactate transporter (MCT1), and MSDC-0160 for the mitochondrial pyruvate carrier (MPC). GAPDH knockdown was performed using siRNA, and cell viability was assessed *via* LDH release assays. For *in vivo* studies, wild-type C57BL/6J mice received intravitreal injections of heptelidic acid, while control mice received the vehicle (dimethyl sulfoxide). Retinal vascular permeability was assessed by fluorescein angiography (FA) and retinal albumin leakage. The most significant decrease in electrical resistance and increase in capacitance of HRECs were observed following the dose-dependent inhibition of GAPDH and the resulting reduction in 1,3-bisphosphoglycerate (1,3-BPG) and NADH by heptelidic acid. LDH level analysis at 24 to 48 h post-treatment with heptelidic acid (1 and 10 μM) showed no significant difference compared to controls, indicating that the observed disruption of HREC functionality was not due to cell death. Supporting these findings, inhibition of downstream glycolytic steps that result in the accumulation of 1,3-BPG and NADH, such as treatment with NG-52 for PGK or shikonin for PKM, led to a significant increase in electrical resistance and a decrease in cell capacitance. Furthermore, GAPDH knockdown *via* siRNA also led to a significant decrease in cellular resistance in HRECs. *In vivo*, FA imaging demonstrated that intravitreal injection of heptelidic acid led to significant retinal vascular leakage, as further supported by increased albumin extravasation in treated eyes. Conversely, pharmacological inhibition of other lower glycolytic components, including LDH, MCT, and MPC, did not significantly alter HREC barrier function or spreading behavior. This study highlights the distinct roles of lower glycolytic components in regulating HREC functionality. GAPDH and its downstream products (1,3-BPG and NADH) are shown to play a pivotal role in maintaining barrier integrity and promoting HREC adhesion and spreading. These findings guide the development of targeted interventions that modulate HREC bioenergetics to treat endothelial dysfunction in various retinal disorders, while minimizing potential adverse effects on healthy endothelial cells.

Under physiological conditions, the retinal endothelium plays a key role in maintaining the integrity of the inner blood-retinal barrier (iBRB), preventing the entry of fluids and solutes from the bloodstream into the inner retina (1). In addition to its barrier function, the retinal endothelium is also responsible for cell adhesion to the extracellular matrix, a critical process that stabilizes endothelial cells within the vascular structure. Cell adhesion to the extracellular matrix promotes the subsequent process of cell spreading, which is essential for maintaining endothelial cell morphology, motility, and overall barrier function (2, 3). Disruptions in these processes can impair endothelial cell function, weaken the iBRB, and contribute to various retinal vascular diseases. For instance, in nonproliferative diabetic retinopathy, the most common form of diabetic retinopathy, early stages are characterized by endothelial cell damage, increased iBRB permeability, and impaired cell adhesion and spreading. As the condition worsens, persistent leakage from damaged blood vessels results in the formation of large cystoid spaces in the macula leading to diabetic macular edema and eventual vision loss (4, 5). As a result, significant research has been dedicated to understanding the mechanisms that preserve endothelial cell adhesion, spreading, and barrier function, with glucose homeostasis emerging as a critical area of interest.

Glucose homeostasis in endothelial cells is maintained through the coordinated interaction of glycolysis, the Krebs cycle, and mitochondrial oxidative phosphorylation (OxPhos). Endothelial cells, however, preferentially rely on glycolysis under normal conditions to minimize oxidative stress (6). Glycolysis, one of the most conserved metabolic pathways across prokaryotic and eukaryotic cells, is divided into two parts (Fig. 1): the upper glycolysis, which invests ATP to break down glucose into two trioses—glyceraldehyde-3-phosphate and dihydroxyacetone phosphate—and the lower glycolysis, which generates pyruvate and produces a net gain of ATP (7). Although retinal endothelial cells depend heavily on glycolysis (8), the specific roles of individual glycolytic enzymes in maintaining the integrity of retinal endothelium barrier remain unclear. Our previous study has examined the role of upper glycolytic components in supporting various aspects of the human retinal endothelial cell (HREC) barrier functionality, finding that disruption of these components can differentially affect the barrier (9). However, the contributions of lower glycolytic enzymes have yet to be fully investigated.

**Figure 1:**
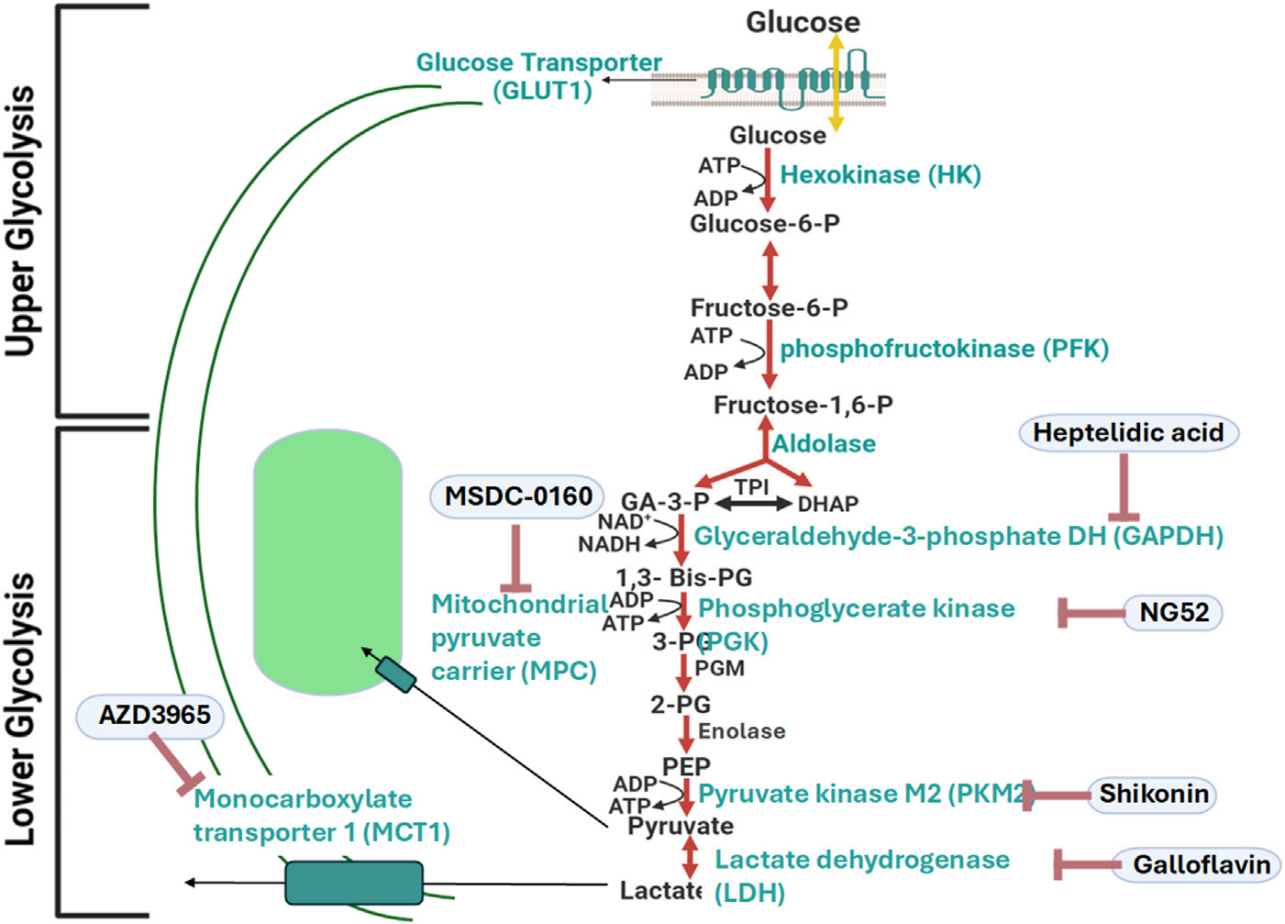
**Schematic representation of glycolysis in human retinal endothelial cells, delineating the division between upper and lower glycolysis.** The inhibitors targeting lower glycolytic components—heptelidic acid, NG-52, shikonin, galloflavin, AZD3965, and MSDC-0160—are labeled in *blue*. 1,3-Bis-PG, 1,3-bisphosphoglycerate; 2-PG, 2-phosphoglycerate; 3-PG, 3-phosphoglycerate; DHAP, dihydroxyacetone phosphate; GA-3-P, glyceraldehyde-3-phosphate; PEP, phosphoenolpyruvate; PGM, phosphoglycerate mutase; TPI, triose phosphate isomerase.

The key steps of lower glycolysis that lead to pyruvate production involve reactions catalyzed by three essential enzymes: GAPDH, phosphoglycerate kinase (PGK), and pyruvate kinase M (PKM). GAPDH, the initial enzyme in this pathway, is critical for converting glyceraldehyde-3-phosphate into 1,3-bisphosphoglycerate (1,3-BPG) while reducing NAD^+^ to NADH. This is the only step in the glycolytic pathway in which NAD^+^ is converted into NADH (10). This reaction not only facilitates the production of ATP but also plays a significant role in controlling the flux of metabolism from upper to lower glycolysis (7). The discovery of heptelidic acid, a pharmacological inhibitor of GAPDH, has provided a valuable tool for investigating the enzyme's role in various cellular processes (11). Despite the importance of GAPDH in glycolysis, its specific contributions to the regulation of cellular functions in HRECs are largely unexplored.

PGK is the second enzyme in lower glycolysis, catalyzing the conversion of 1,3-BPG and ADP to 3-phosphoglycerate and ATP. Its crystal structure reveals a dimeric protein comprising a large α/β domain and a smaller β-barrel domain, with the active site situated in a cleft between these domains (12, 13). The large domain binds substrates and facilitates the phosphorylation reaction, while the β-barrel domain stabilizes the dimer and aids in the transfer of the phosphoryl group (12, 13). NG52, a potent and selective inhibitor of PGK, binds to the ATP-binding pocket, blocking substrate interaction and preventing the conversion of 1,3-BPG (14). This inhibition results in the accumulation of 1,3-BPG and disrupting the enzyme's catalytic activity. Although PGK is known to be involved in endothelial cell and tumor cell angiogenesis through increasing angiostatin (15, 16, 17), its role in maintaining the barrier integrity of HRECs remains to be fully elucidated.

The final enzyme in lower glycolysis is PKM, which is crucial for regulating glycolysis in endothelial cells. PKM catalyzes the conversion of phosphoenolpyruvate into pyruvate, a step that generates ATP and drives glycolytic flux (6, 18). Shikonin is recognized as an inhibitor of PKM, binding directly to the enzyme and disrupting its catalytic activity, which leads to a reduction in pyruvate and accumulation of upstream intermediates, including 1,3-BPG and NADH (19). The inhibition of PKM by shikonin influences various glycolysis-dependent cellular processes, leading to altered metabolic flux. However, the precise role of PKM in maintaining the barrier integrity and cellular behavior of HRECs remains poorly understood.

After pyruvate is formed in the final steps of glycolysis, it can follow one of the two key metabolic pathways, depending on the cell's metabolic needs and oxygen availability. In the first pathway, pyruvate is transported into the mitochondria *via* the mitochondrial pyruvate carrier (MPC), where it is converted into acetyl-CoA by the pyruvate dehydrogenase complex. Acetyl-CoA then enters the citric acid cycle, producing NADH and FADH2, which fuel OxPhos for efficient ATP production (20, 21). In the second pathway, under hypoxic conditions or when glycolytic activity is high, pyruvate is reduced to lactate by lactate dehydrogenase (LDH) in the cytoplasm. This reaction regenerates NAD+ from NADH, which is crucial for sustaining glycolysis. The lactate produced is subsequently exported from the cell *via* a monocarboxylate transporter (MCT)1, preventing intracellular acidification and maintaining metabolic balance (22, 23). Both pathways are vital for regulating energy metabolism and ensuring proper cellular function, especially in cells like HRECs, which depend on a dynamic interplay between mitochondrial respiration and glycolysis to meet their physiological demands. However, the relative contributions of these pathways to maintaining HREC barrier integrity and cellular behavior under normal conditions are not fully understood.

To address these knowledge gaps, we employed the electric cell-substrate impedance sensing (ECIS) system to measure, in real-time, changes in barrier integrity and cellular spreading of HRECs in response to lower glycolytic pharmacological inhibitors. ECIS technology applies a constant alternating current to measure voltage changes across electrodes where cells are plated, enabling the calculation of impedance, from which barrier resistance and cell capacitance are derived. Resistance measured at low frequencies (*e.g.*, 4000 Hz) reflects paracellular barrier integrity, as intact tight junctions restrict electron flow, whereas a decrease in resistance indicates weakened cell–cell junctions and barrier dysfunction. Conversely, capacitance at high frequencies (*e.g.*, 64,000 Hz) corresponds to cell spreading and membrane interactions with the substrate. As cells spread over the electrode surface, they increase membrane coverage, reducing the capacitance signal. Conversely, a higher capacitance suggests reduced cell-substrate coverage, indicating impaired adhesion, morphological changes, or cytoskeletal alterations (24, 25). Our previous ECIS experiments demonstrated that disrupting various components of mitochondrial OxPhos and upper glycolysis affected the HREC functions beyond their role in ATP production (9, 26). In this study, we aimed to evaluate the relative contributions of lower glycolytic components to HREC functionality, specifically focusing on barrier integrity and cell spreading over the substrate, using ECIS technology.

## Results

### Real-time monitoring of the effect of lower glycolytic components on HREC functions using ECIS

**Figure 2:**
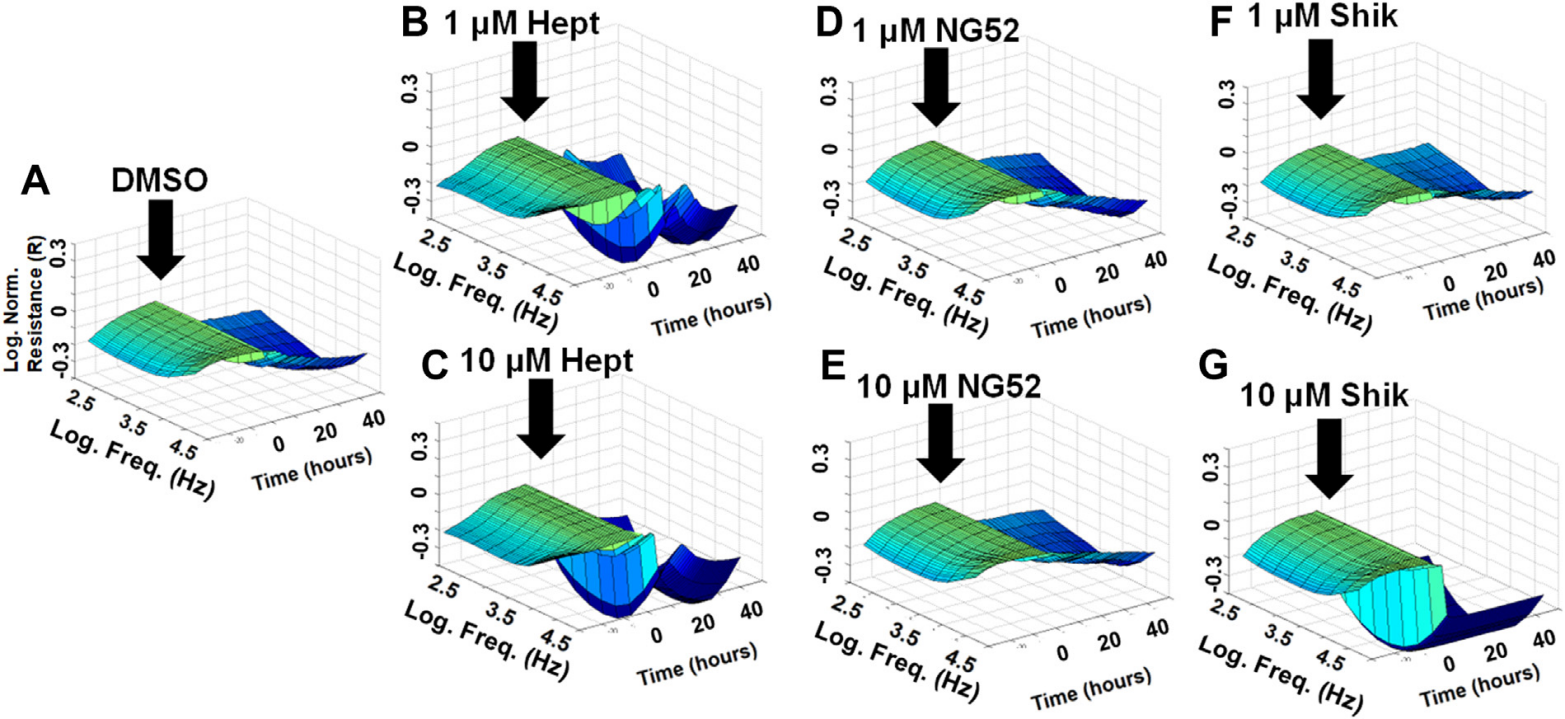
**Resistance analysis of the effects of lower glycolytic inhibitors on HREC barrier integrity using ECIS technology.** Three-dimensional plots depict the log of normalized resistance (R) over time and the log of the alternating current (AC) frequencies. *A*, HRECs treated with vehicle (DMSO). *B*, HRECs treated with 1 μM GAPDH inhibitor (heptelidic acid, Hept). *C*, HRECs treated with 10 μM GAPDH inhibitor (Hept). *D*, HRECs treated with 1 μM PGK inhibitor (NG-52). *E*, HRECs treated with 10 μM PGK inhibitor (NG-52). *F*, HRECs treated with 1 μM PKM2 inhibitor (shikonin, Shik). *G*, HRECs treated with 10 μM PKM2 inhibitor (Shik). Treatments were applied at t = 0 h after HRECs formed a confluent monolayer. The resistance value at t = 0 h (R_0_) was used to normalize all subsequent impedance measurements (R_t_) as a ratio (R_t_/R_0_). Real-time resistance measurements were conducted across AC frequencies ranging from 250 to 64,000 Hz. Freq, frequency; Hept, heptelidic acid; Norm, normalized; R, resistance; R_t_, resistance at time t; R_0_, resistance at time 0; Shik, shikonin.

**Figure 3:**
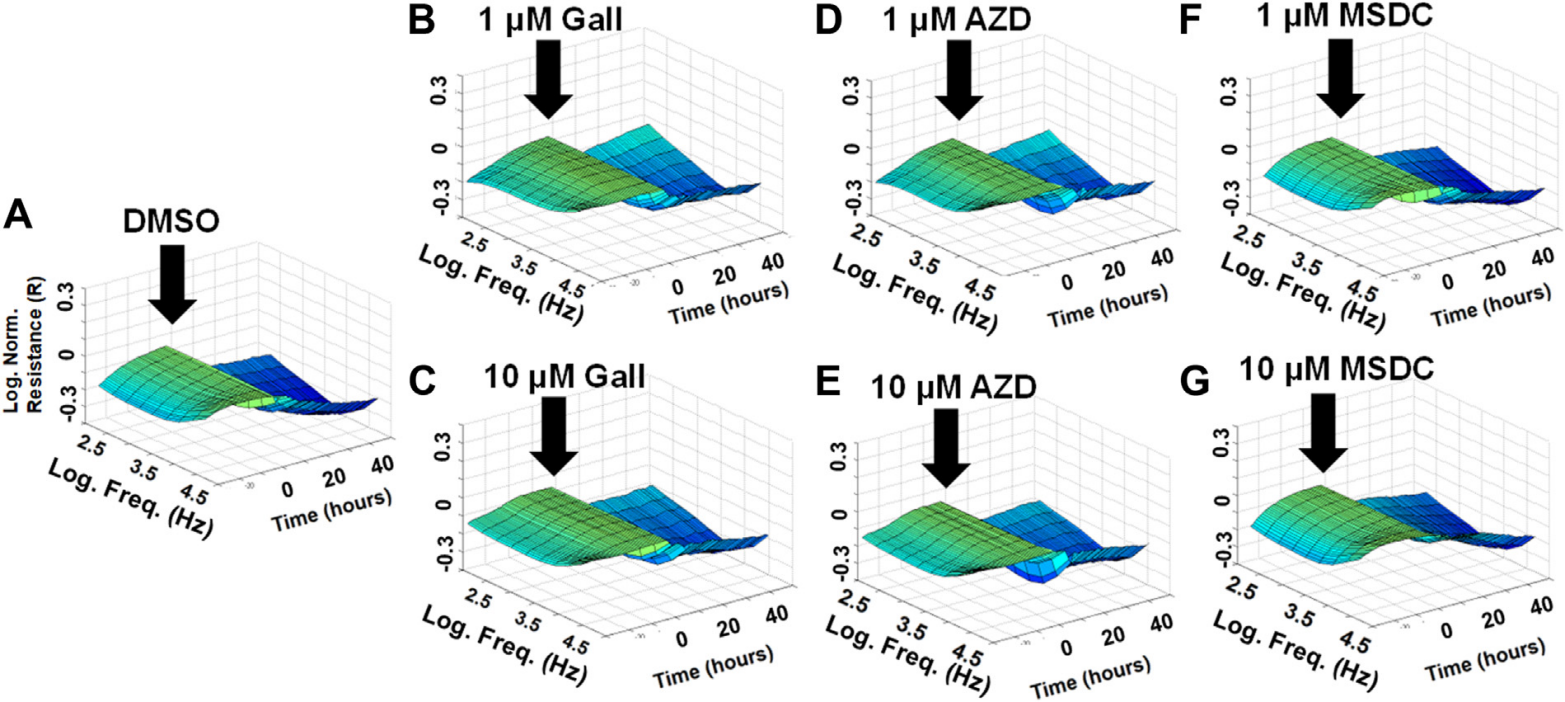
**Resistance analysis of the effects of other lower glycolysis-related component inhibitors on HREC barrier function using ECIS technology.** Three-dimensional plots depict the log of normalized resistance (R) over time and the log of AC frequencies. *A*, HRECs treated with vehicle (DMSO). *B*, HRECs treated with 1 μM LDH inhibitor (galloflavin, Gall). *C*, HRECs treated with 10 μM LDH inhibitor (Gall). *D*, HRECs treated with 1 μM MCT1 inhibitor (AZD3965, AZD). *E*, HRECs treated with 10 μM MCT1 inhibitor (AZD). *F*, HRECs treated with 1 μM MPC inhibitor (MSDC-0160, MSDC). *G*, HRECs treated with 10 μM MPC inhibitor (MSDC). Treatments were applied at t = 0 h after HRECs formed a confluent monolayer. The resistance value at t = 0 h (R_0_) was used to normalize all subsequent resistance measurements (R_t_) as a ratio (R_t_/R_0_). Real-time resistance measurements were conducted across the log of AC frequencies ranging from 250 to 64,000 Hz. AZD, AZD3965; Freq, frequency; Gall, galloflavin; MSDC, MSDC-0160; Norm, normalized; R_0_, resistance at time 0; R, resistance; R_t_, resistance at time t.

Since the impaired barrier function of HRECs plays a critical role in the development of several retinal diseases (2), we used ECIS technology to investigate the differential roles of lower glycolytic components in maintaining HREC barrier integrity. Various lower glycolytic inhibitors were employed to assess their impact on this function, including dimethyl sulfoxide (DMSO) as a vehicle control (Figs. 2*A* and 3*A*), heptelidic acid for inhibiting GAPDH (Fig. 2, *B* and *C*), NG52 for inhibiting PGK (Fig. 2, *D* and *E*), shikonin for inhibiting PKM (Fig. 2, *F* and *G*), galloflavin for inhibiting LDH (Fig. 3, *B* and *C*), AZD3965 for inhibiting MCT1 (Fig. 3, *D* and *E*), and MSDC-0160 for inhibiting MPC (Fig. 3, *F* and *G*). Two concentrations of these inhibitors were applied to HRECs after reaching a confluent monolayer, as indicated by a plateau in electrical resistance along the y-axis in the 3D model (black arrows in Figs. 2 and 3). Following the addition of the inhibitors, barrier integrity was continuously monitored in real-time by measuring electrical resistance over time (displayed on the z-axis) across a frequency range of 250 to 64,000 Hz (shown on the x-axis). The 3D visual models (Figs. 2 and 3) demonstrated noticeable variations in electrical resistance corresponding to the inhibition of specific lower glycolytic components. Notably, inhibition of GAPDH using heptelidic acid resulted in a clear dose-dependent decrease in HREC resistance, suggesting a critical role for this enzyme in maintaining barrier function. Consistently, inhibition of downstream glycolytic steps that result in the accumulation of 1,3-BPG and NADH, using NG-52 (10 μM) to inhibit PGK and shikonin (1 μM) to inhibit PKM, led to a notable increase in electrical resistance. In contrast, no obvious changes in electrical resistance were observed when using galloflavin (LDH inhibitor), AZD3965 (MCT1 inhibitor), or MSDC-0160 (MPC inhibitor) at either 1 μM or 10 μM, compared to the DMSO-treated control group (Fig. 3). These results emphasize the distinct roles that specific lower glycolytic components play in regulating HREC functions. The following experiments have been conducted to better understand these roles.

### The effect of GAPDH inhibition on HREC functions

**Figure 4:**
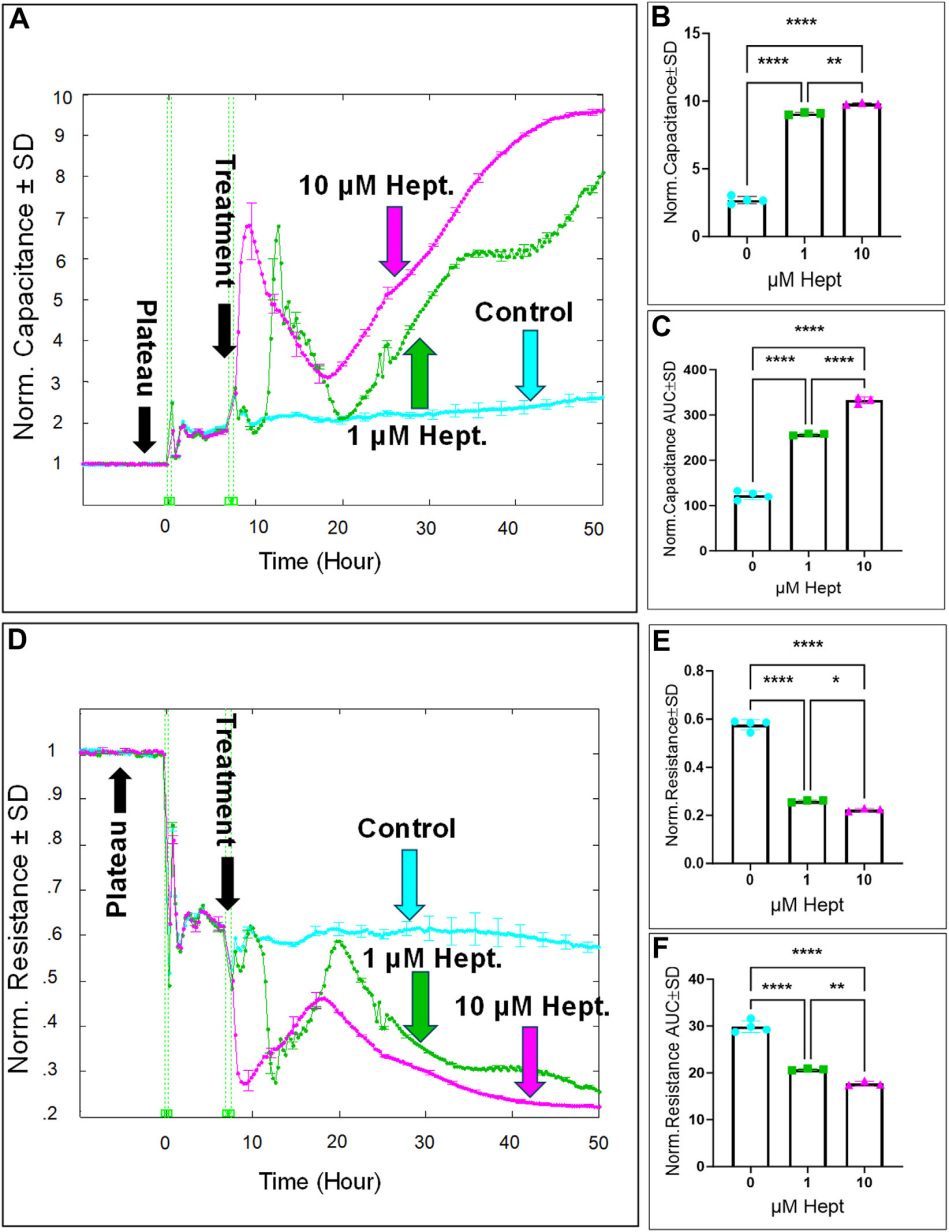
**Real-time measurement of the total capacitance and resistance across HRECs treated with GAPDH inhibitor (Hept).***A* and *D*, plots of normalized capacitance and resistance, respectively, across HRECs versus time measured at an AC frequency of 64,000 for capacitance and 4000 Hz for resistance and HRECs treated with heptelidic acid (1 μM & 10 μM). The measurement of capacitance and resistance started from the time HRECs were placed on the ECIS electrode to the endpoint of the experiment (t = 50 h). Normalization was performed with the capacitance and resistance values measured at t = 0 h. *B* and *E*, bar graph representation of the normalized capacitance and resistance for control and HRECs treated with heptelidic acid (1 μM & 10 μM) at the endpoint of the experiment (t = 50 h). *C* and *F*, bar graph representation of the areas under the normalized capacitance/resistance curve for control and HRECs treated with heptelidic acid (1 μM & 10 μM) at the time interval t = 0 to 50 h. Statistical comparisons were performed using one-way ANOVA. The false discovery rate (FDR) was controlled using the two-stage linear step-up procedure of Benjamin, Krieger, and Yekutieli, with a threshold of <0.05. Significant changes are specified by *p* values of symbol ∗∗∗∗<0.0001, ∗∗<0.01, ∗<0.05; n = 3 to 4 biological replicates for each group. AUC, area under the curve; Norm, normalized.

**Figure 5:**
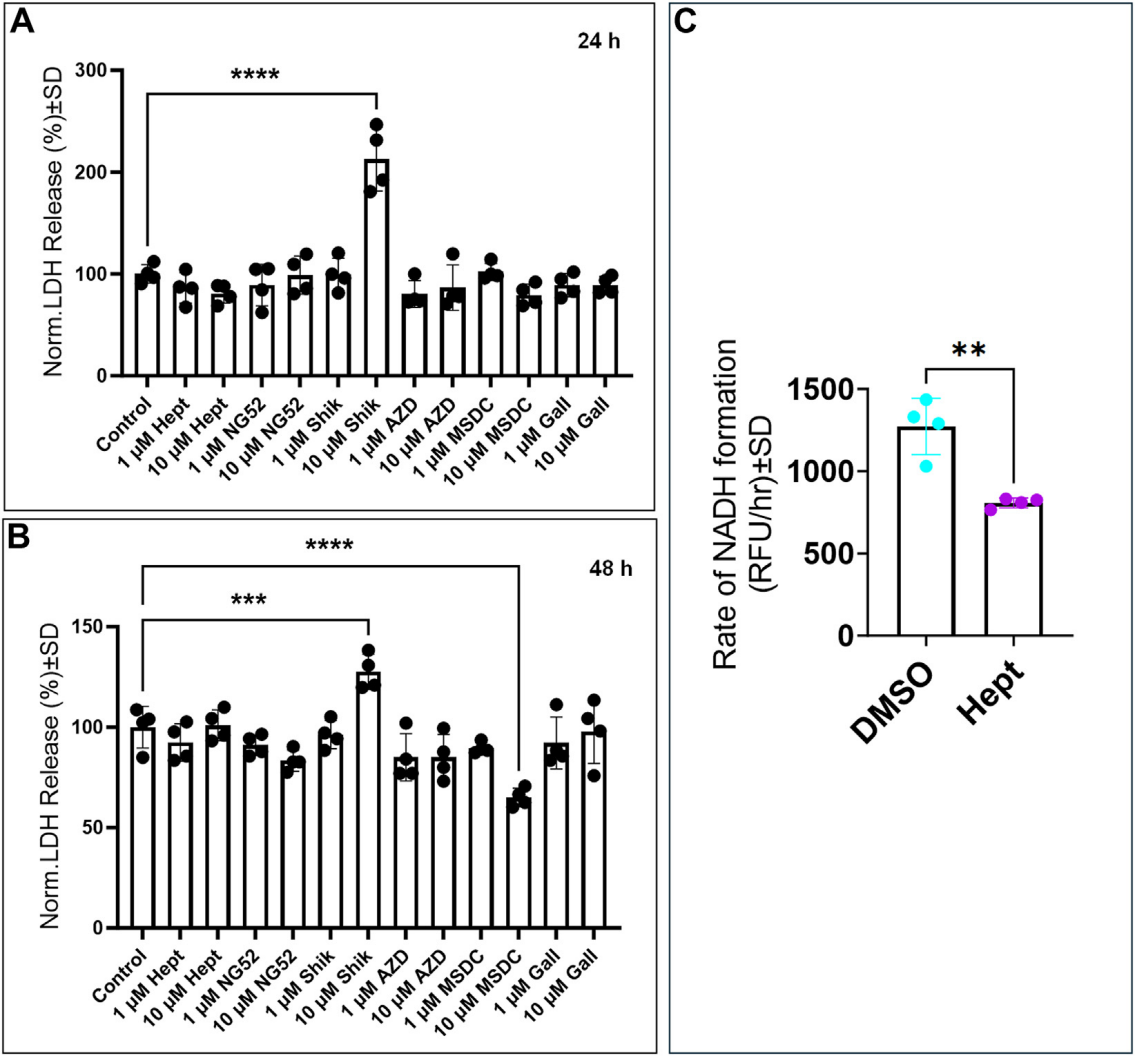
**Effects of glycolytic inhibitors on HRECs viability and rate of NADH formation.** LDH assays were performed at 24 h and 48 h for controls and cells treated with different lower glycolytic inhibitors. HRECs death was monitored by measuring the amount of lactate dehydrogenase (LDH) released in the culture media. *A*, effect of different glycolytic inhibitors on HRECs viability at 24 h and (*B*) at 48 h. Only shikonin at a concentration of 10 μM significantly induced HRECs death at 24 h and 48 h as compared to control. *C*, rate of NADH formation of HRECs treated with DMSO and HRECs treated with heptelidic acid. Statistical comparisons were performed using one-way ANOVA and independent *t* test analysis. The false discovery rate (FDR) was controlled using the two-stage linear step-up procedure of Benjamin, Krieger, and Yekutieli, with a threshold of <0.05. Significant changes are specified by *p* values of symbol ∗∗∗∗<0.0001, ∗∗∗<0.001, ∗∗<0.01; n = 4 biological replicates/group.

Cell spreading and the maintenance of barrier integrity are crucial functions of HRECs that contribute to the stability of the iBRB. These characteristics can be continuously monitored in real-time by measuring capacitance and resistance, respectively. Capacitance reflects how well cells spread over the substrate, while resistance is indicative of cell–cell junction strength, cell morphology, and cell–matrix adhesion (27). To investigate the role of GAPDH in HREC spreading, we monitored capacitance across HREC monolayers at a frequency of 64,000 Hz, identified in our previous research (28) as optimal for measuring the maximum extent of cell spreading over the electrode. Since capacitance decreases as cells spread, it was important to assess how GAPDH inhibition affected this process once the cells reached electrical quiescence, defined as the stable minimum plateau in the capacitance curves after the cells had settled on the electrode (black arrow in Figure 4*A*). As shown in Figure 4*A*, HRECs treated with heptelidic acid showed a distinctive pattern, with capacitance rising from baseline to a temporary maximum, slowing for several hours, and then increasing steadily for the remainder of the experiment. At the end of the experiment, heptelidic acid dose-dependently increased capacitance (Fig. 4*B*). Additionally, the area under the curve (AUC) was calculated for each capacitance curve to evaluate the effects of GAPDH inhibition throughout the experiment. As shown in Figure 4*C*, the AUCs differed significantly across the heptelidic acid–treated groups, indicating that GAPDH inhibition significantly impacted HREC capacitance and thus cell spreading in a dose-dependent manner. Notably, the impact of heptelidic acid on HREC spreading occurred without compromising cell viability (Fig. 5). Additionally, at 24 and 48 h intervals (Fig. 5, *A* and *B*), only shikonin (10 μM) caused a significant increase in LDH release, indicating cytotoxicity. In contrast, NG-52 (1 and 10 μM), shikonin (1 μM), galloflavin (1 and 10 μM), AZD3965 (1 and 10 μM), and MSDC-0160 (1 and 10 μM) showed no cytotoxic effects. To confirm the specificity of heptelidic acid as a GAPDH inhibitor, we measured NADH production as a metabolic readout of GAPDH enzymatic activity. As shown in Figure 5*C*, heptelidic acid treatment led to a significant reduction in NADH levels, confirming its role in disrupting GAPDH-dependent metabolic activity.

**Figure 6:**
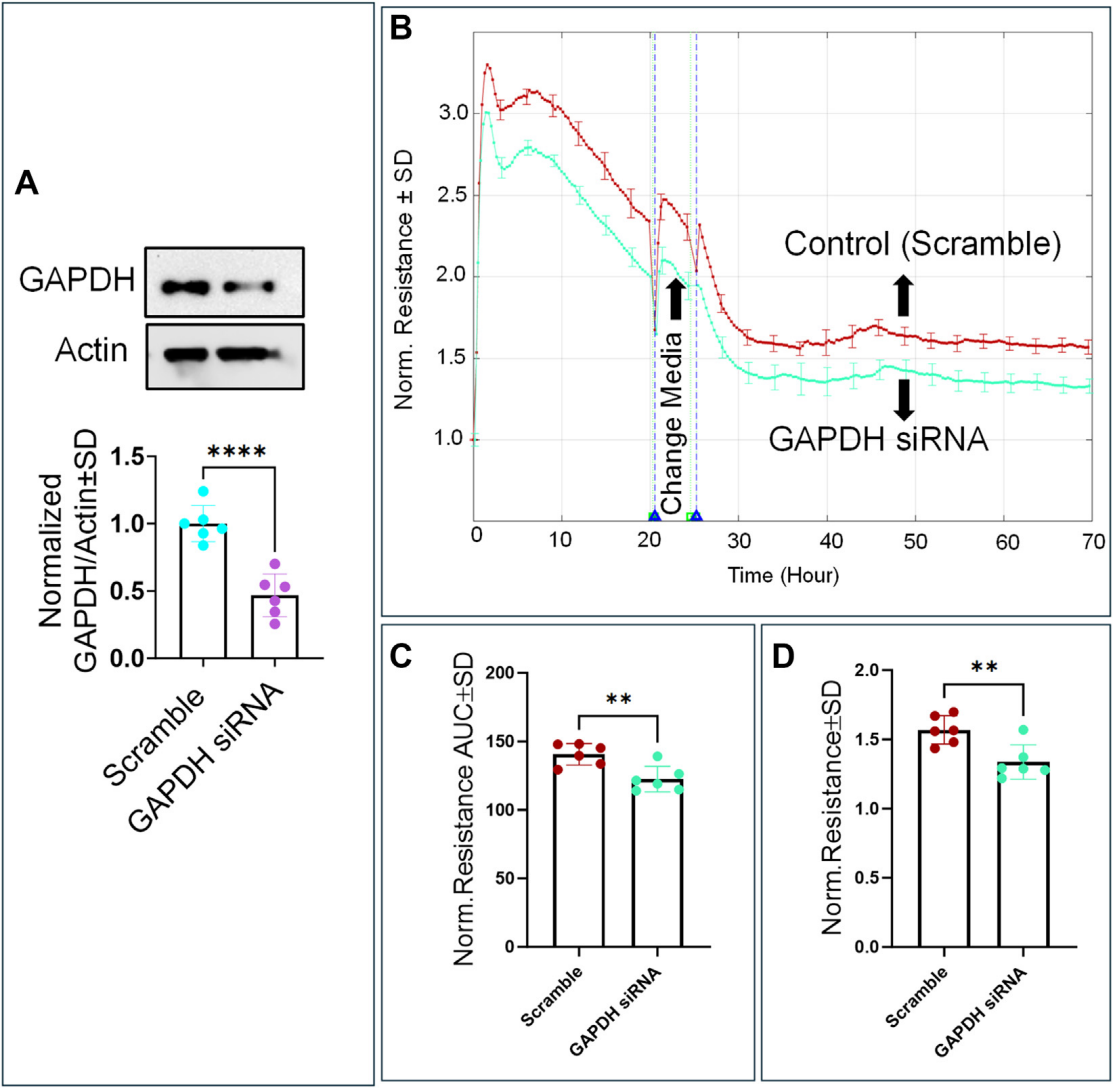
**Real-time measurement of the resistance across HRECs treated with GAPDH siRNA and measurement of GAPDH levels in HRECs treated with GAPDH siRNA.***A*, representative immunoblots and a figure for GAPDH levels normalized to actin in scramble and HRECs treated with GAPDH siRNA. *B*, plots of normalized resistance versus time across HRECs treated with DsiRNA (scramble) and HRECs treated with GAPDH siRNA. The measurement of resistance started from the time HRECs were placed on the ECIS electrode to the endpoint of the experiment. *C*, bar graph representation of the areas under the normalized resistance curve for scramble and HRECs treated with GAPDH siRNA at time interval t = 0–70 h. *D*, bar graph representation of the normalized resistance for scramble and HRECs treated with GAPDH siRNA at the endpoint of the experiment. Statistical comparisons were performed using independent *t* test analysis. Significant changes are specified by *p* values of symbol ∗∗∗∗<0.0001, ∗∗<0.01; n = 6 biological replicates for each group.

Next, we investigated the role of GAPDH in maintaining HREC barrier integrity by pharmacologically inhibiting it with heptelidic acid, followed by measuring resistance across HREC monolayers at an optimal frequency of 4000 Hz, which corresponds to the maximal resistance determined from our previous publication (28). As shown in Figure 4*D*, treatment with both 1 μM and 10 μM heptelidic acid resulted in a dose-dependent reduction in resistance, followed by a transient recovery, and then a further sustained decline compared to the control group. These dose-dependent changes in resistance were observed both at the experiment’s endpoint (Fig. 4*E*) and throughout its duration (Fig. 4*F*), suggesting that GAPDH inhibition irreversibly impairs HREC barrier function in a dose-dependent manner. To further evaluate the role of GAPDH in barrier integrity, we knocked down GAPDH using siRNA transfection. As shown in Figure 6*A*, GAPDH knockdown resulted in a significant reduction in protein levels, which was associated with a decrease in HREC resistance compared to the scramble-treated control (Fig. 6*B*). This reduction in barrier resistance was sustained throughout the experiment as well as at its endpoint (Fig. 6, *C* and *D*, respectively), indicating a consistent impairment of HREC barrier function following GAPDH knockdown.

**Figure 7:**
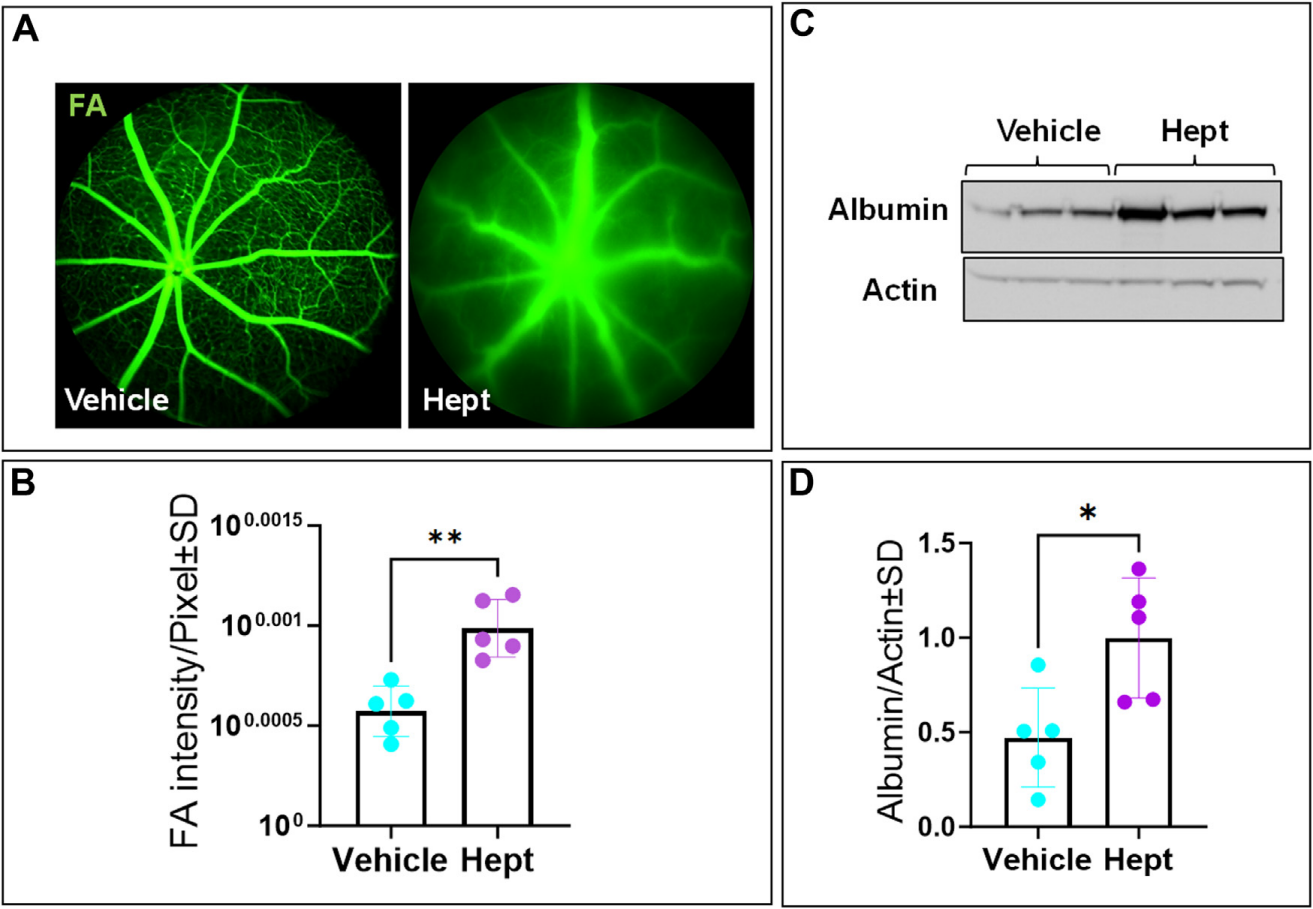
**The effect of inhibiting GAPDH with heptelidic acid using fluorescein angiography *invivo*.***A* and *B*, injection of mouse eyes with heptelidic acid led to visible leakage of the retinal vessels on FA compared to vehicle injection and increased FA intensity. *C* and *D*, representative immunoblots and a figure showing albumin/actin ratio in eyes treated with heptelidic acid compared to vehicle. Statistical comparisons were performed using independent *t* test analysis. Significant changes are specified by *p* values of symbol ∗∗<0.01, ∗<0.05; n = 5 biological replicates/group.

Given that both pharmacological inhibition and genetic knockdown of GAPDH compromised endothelial barrier integrity *in vitro*, we next sought to determine whether these effects translate to an *in vivo* setting. To validate the effects of GAPDH inhibition *in vivo*, we performed fluorescein angiography (FA) following intravitreal injection of heptelidic acid (10 μM). As shown in Figure 7*A*, eyes treated with heptelidic acid exhibited visible leakage of retinal vessels on FA, accompanied by a significant increase in FA intensity (Fig. 7*B*). These findings were further supported by elevated retinal albumin in heptelidic acid–treated eyes compared to vehicle-treated controls (Fig. 7, *C* and *D*), confirming that GAPDH inhibition disrupts retinal vascular integrity *in vivo*, consistent with our *in vitro* findings.

### The effect of PGK and PKM inhibition on HREC functions

**Figure 8:**
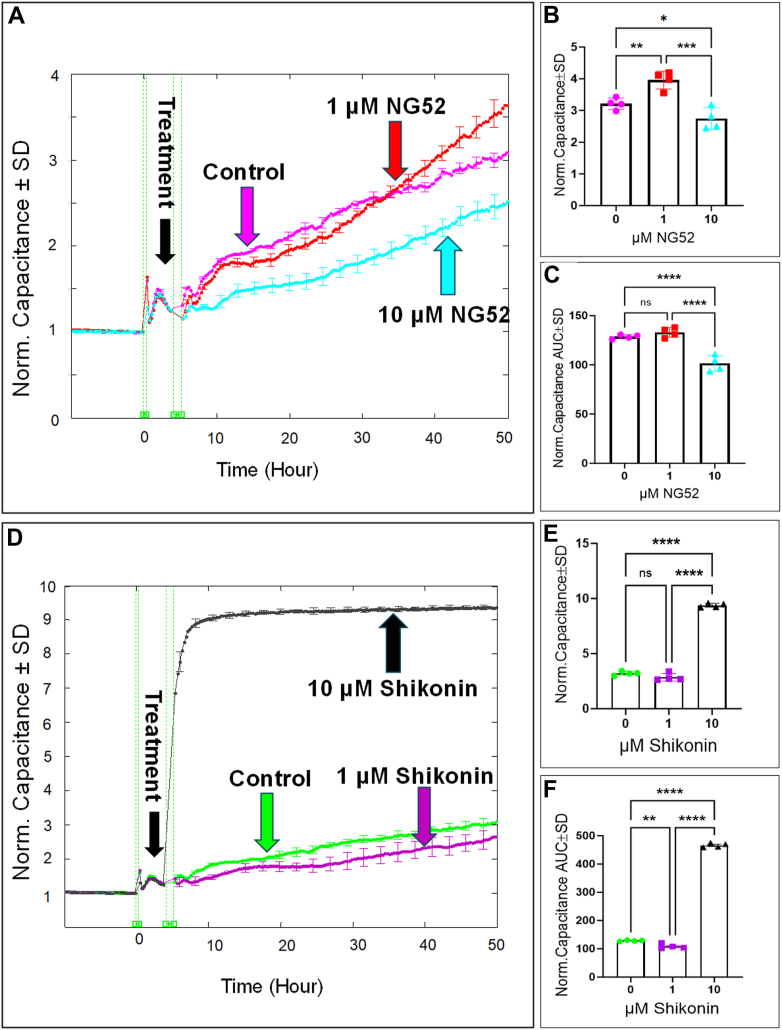
**Real-time measurement of the total capacitance across HRECs treated with PGK1 inhibitor (NG52) and PKM2 inhibitor (Shikonin).***A* and *D*, plots of normalized capacitance across HRECs versus time measured at an AC frequency of 64,000 Hz for HRECs treated with (1 μM & 10 μM) of NG52 or shikonin, respectively. Normalization was performed with the capacitance values measured at t = 0 h. *B* and *E*, bar graph representation of the normalized capacitance for control and HRECs treated with NG52 or shikonin (1 μM & 10 μM) at the endpoint of the experiment (t = 50 h). *C* and *F*, bar graph representation of the areas under the normalized capacitance curve for control and HRECs treated with NG52 or shikonin (1 μM & 10 μM) at the time interval t = 0 to 50 h. Statistical comparisons were performed using one-way ANOVA. The false discovery rate (FDR) was controlled using the two-stage linear step-up procedure of Benjamin, Krieger, and Yekutieli, with a threshold of <0.05. Significant changes are specified by *p* values of symbol ∗∗∗∗<0.0001, ∗∗∗<0.001, ∗∗<0.01, ∗<0.05; n = 4 biological replicates for each group. AUC, area under the curve; Norm, normalized; ns, no significance.

**Figure 9:**
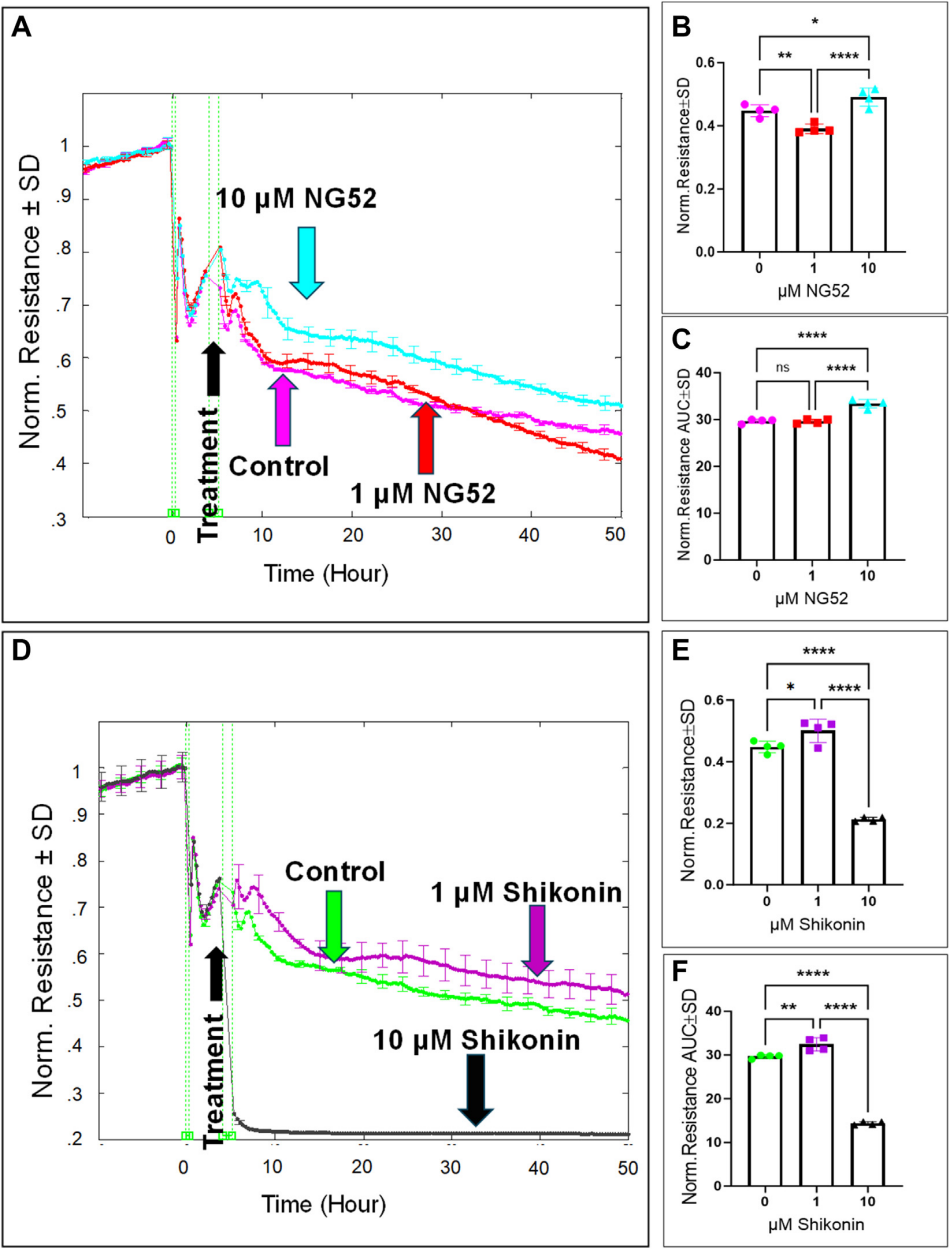
**Real-time measurement of the total resistance across HRECs treated with PGK1 inhibitor (NG52) and PKM2 inhibitor (Shikonin).***A* and *D*, plots of normalized resistance across HRECs versus time measured at an AC frequency of 4000 Hz for HRECs treated with (1 μM & 10 μM) of NG52 or shikonin, respectively. Normalization was performed with the resistance values measured at t = 0 h. *B* and *E*, bar graph representation of the normalized resistance for control and HRECs treated with NG52 or shikonin at the endpoint of the experiment (t = 50 h). *C* and *F*, bar graph representation of the areas under the normalized resistance curve for control and HRECs treated with NG52 or shikonin at the time interval t = 0 to 50 h. Statistical comparisons were performed using one-way ANOVA. The false discovery rate (FDR) was controlled using the two-stage linear step-up procedure of Benjamin, Krieger, and Yekutieli, with a threshold of <0.05. Significant changes are specified by *p* values of symbol ∗∗∗∗<0.0001, ∗∗<0.01, ∗<0.05; n = 4 biological replicates for each group. AUC, the area under the curve; Norm, normalized.

We next examined the effect of inhibiting downstream glycolytic steps, following GAPDH, that lead to the accumulation of 1,3-BPG, using NG52 for PGK inhibition and shikonin for PKM inhibition on the behavior of HRECs. First, we investigated how PGK or PKM inhibition affected HREC spreading by monitoring capacitance across the HREC monolayers at a frequency of 64,000 Hz (Fig. 8, *A* and *D*, respectively). Treatment with NG52 (10 μM) or shikonin (1 μM) resulted in a decrease in capacitance compared to the control group at the end of the experiment, indicating enhanced cell spreading (Fig. 8, *B* and *E*, respectively). To assess the cumulative effect of PGK or PKM inhibition on cell spreading, we calculated the AUC for capacitance throughout the experimental period. The AUC for the NG52-treated (10 μM) and shikonin-treated (1 μM) groups was significantly lower than that of the control group, further emphasizing the positive effect of NG52 and shikonin on HREC spreading (Fig. 8, *C* and *F*, respectively). Of note, at a concentration of 10 μM, shikonin reached a toxic level, as indicated by a significant increase in LDH release in Figure 5, *A* and *B*, which led to an immediate increase in HREC capacitance (Fig. 8*D*). Next, we evaluated the effect of NG52 and shikonin on HREC barrier integrity by monitoring resistance across the monolayers at a frequency of 4000 Hz. As shown in Figure 9, *A* and *D*, respectively, treatment with NG52 (10 μM) or shikonin (1 μM) resulted in a notable improvement in the resistance curve, indicating enhanced barrier function. This improvement was observed both at the end of the experiment (Fig. 9, *B* and *E*, respectively) and consistently throughout the duration of the experiment (Fig. 9, *C* and *F*, respectively). However, when shikonin reached a toxic concentration (10 μM; Fig. 5), it caused a sharp decline in HREC resistance (Fig. 9*D*). Taken together, these findings suggest that nontoxic inhibition of PGK or PKM, downstream glycolytic steps that lead to the accumulation of 1,3-BPG and NADH, improves both the spreading behavior and barrier integrity of HRECs.

### The effect of inhibiting other lower glycolytic-related components on HREC functions

**Figure 10:**
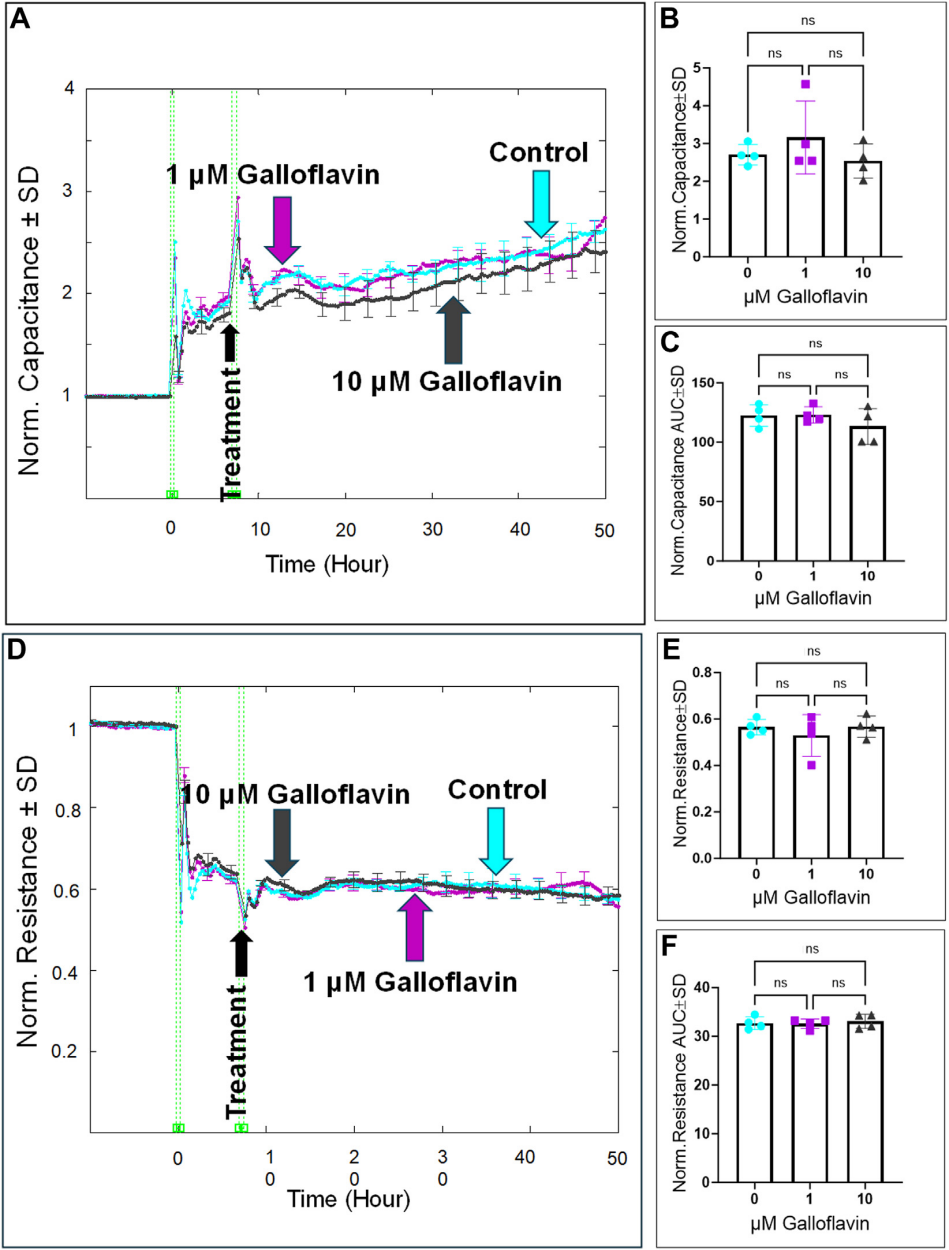
**Real-time measurement of the total capacitance and resistance across HRECs treated with LDH inhibitor (Galloflavin).***A* and *D*, plots of normalized capacitance and resistance, respectively, across HRECs versus time, measured at an AC frequency of 64,000 Hz for capacitance and 4000 Hz for resistance, for control and HRECs treated with galloflavin (1 μM & 10 μM). Normalization was performed with the capacitance and resistance values measured at t = 0 h. *B* and *E*, bar graph representation of the normalized capacitance and resistance at the endpoint of the experiment (t = 50 h) for control and HRECs treated with galloflavin (1 μM & 10 μM). *C* and *F*, bar graph representation of the areas under the normalized capacitance/resistance curve for control and HRECs treated with galloflavin (1 μM & 10 μM) at the time interval t = 0 to 50 h. Statistical comparisons were performed using one-way ANOVA. The false discovery rate (FDR) was controlled using the two-stage linear step-up procedure of Benjamin, Krieger, and Yekutieli, with a threshold of <0.05. n = 4 biological replicates for each group. AUC, the area under the curve; Norm, normalized; ns, no significance.

To explore the role of other lower glycolytic-related components, we first investigated the effects of inhibiting LDH using galloflavin on HREC behaviors. As shown in Figure 10*A*, treatment with either 1 μM or 10 μM galloflavin did not result in any significant changes in the capacitance at a frequency of 64,000 Hz compared to the control. This lack of effect is corroborated by measurements taken at the end of the experiment (Fig. 10*B*) and throughout the duration of the experiment (Fig. 10*C*). Similarly, changes in the resistance over time at a frequency of 4000 Hz also demonstrated no significant effects of LDH inhibition with galloflavin at both concentrations (Fig. 10*D*). This was consistent with the observations at the endpoint of the experiment (Fig. 10*E*) and throughout (Fig. 10*F*).

**Figure 11:**
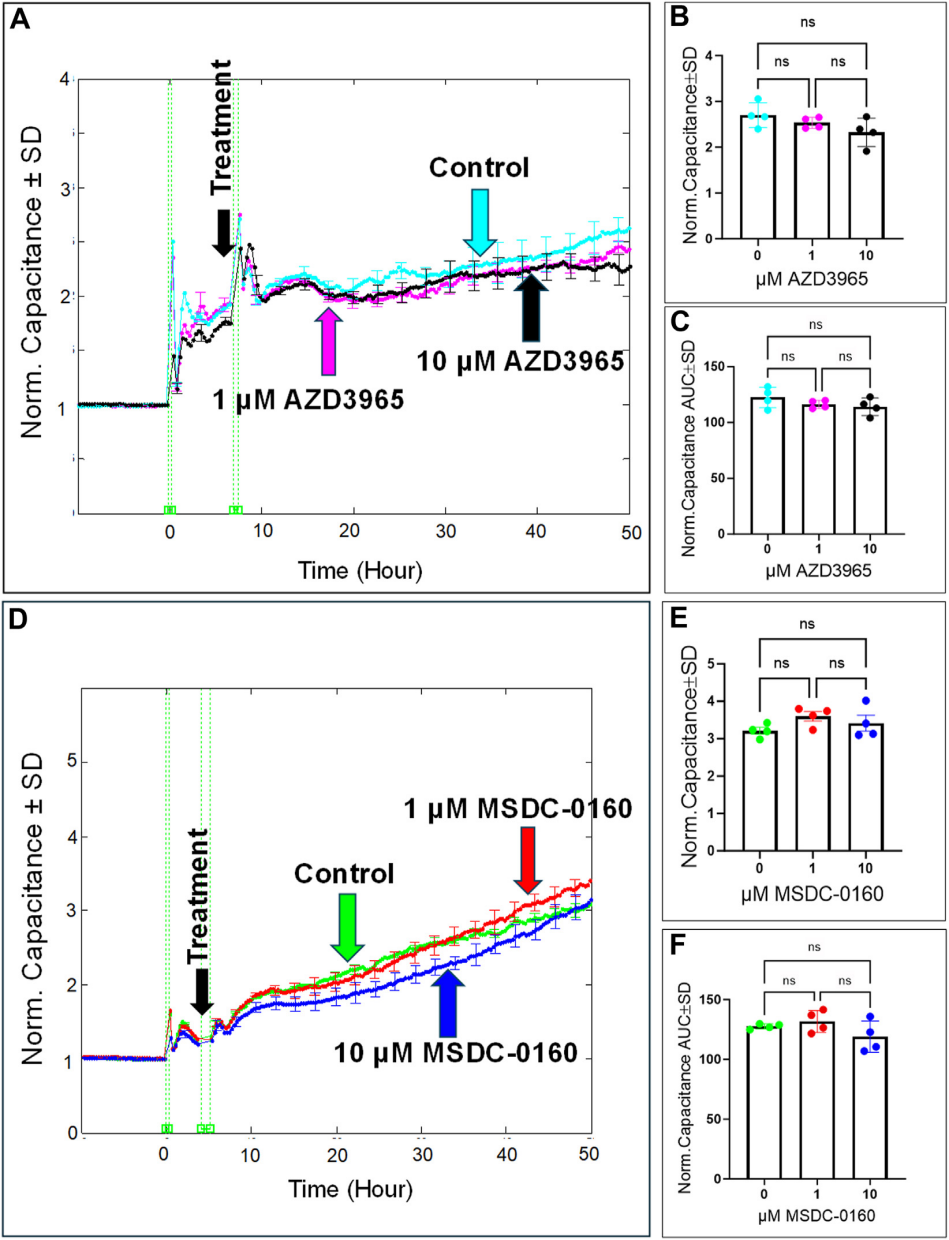
**Real-time measurement of the total capacitance across HRECs treated with MCT1 inhibitor (AZD3965) and MPC inhibitor (MCDC-0160).***A* and *D*, plots of normalized capacitance across HRECs versus time measured at an AC frequency of 64,000 Hz for control and HRECs treated with (1 μM & 10 μM) of AZD3965 or MCDC-0160. Normalization was performed with the capacitance value measured at t = 0 h. *B* and *E*, bar graph representation of the normalized capacitance for control and HRECs treated with (1 μM & 10 μM) of AZD3965 or MSDC-0160 at the endpoint of the experiment (t = 50 h). *C* and *F*, bar graph representation of the areas under the normalized capacitance curve for control and HRECs treated with AZD3965 or MSDC-0160 (1 μM & 10 μM) at the time interval t = 0 to 50 h. Statistical comparisons were performed using one-way ANOVA. The false discovery rate (FDR) was controlled using the two-stage linear step-up procedure of Benjamin, Krieger, and Yekutieli, with a threshold of <0.05. n = 4 biological replicates for each group. AUC, the area under the curve; Norm, normalized; ns, no significance.

**Figure 12:**
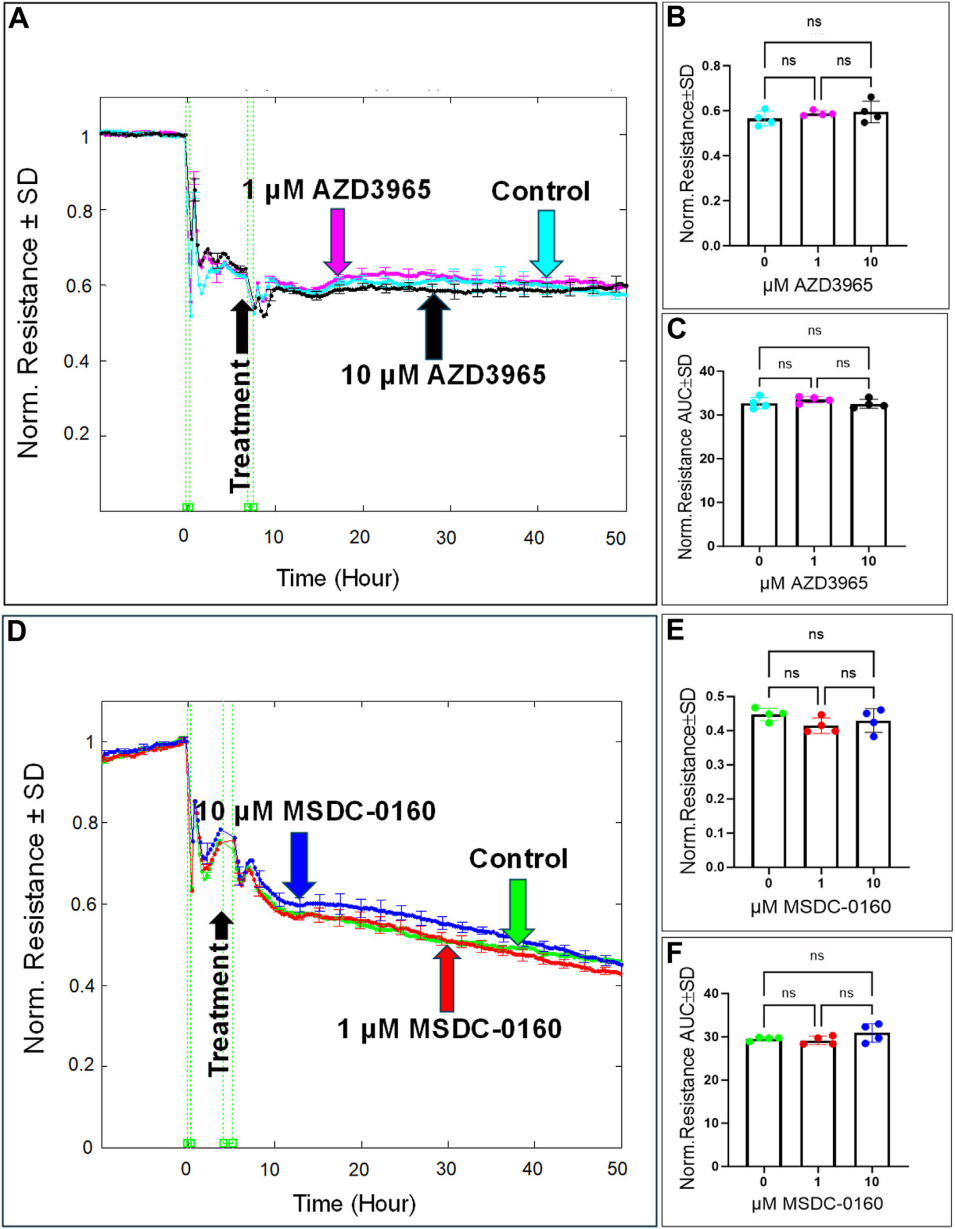
**Real-time measurement of total resistance across HRECs treated with the MCT1 inhibitor (AZD3965) and MPC inhibitor (MSDC-0160).***A* and *D*, plots of normalized resistance over time for control and HRECs treated with AZD3965 or MSDC-0160, respectively, measured at an AC frequency of 4000 Hz. The resistance values were normalized to the initial measurement at t = 0 h. *B* and *E*, bar graphs showing normalized resistance at the experiment endpoint (t = 50 h) for control and treated groups with AZD3965 or MSDC-0160, respectively. *C* and *F*, bar graphs depicting the area under the normalized resistance curve (AUC) for control and treated HRECs with AZD3965 or MSDC-0160, respectively, over the time interval from t = 0 to t = 50 h. Statistical comparisons were performed using one-way ANOVA. The false discovery rate (FDR) was controlled using the two-stage linear step-up procedure of Benjamin, Krieger, and Yekutieli, with a threshold of <0.05. n = 4 biological replicates for each group. AUC, the area under the curve; Norm, normalized; ns, no-significance.

Next, we evaluated the impact of inhibiting the lower glycolytic-related transporters, MCT1 and MPC, on HREC spreading behavior and barrier integrity. Initial measurements of the capacitance over time after MCT1 inhibition with AZD3965 and MPC inhibition with MSDC-0160 revealed no significant changes. Specifically, Figure 11*A* shows that treatment with AZD3965 at 1 μM and 10 μM did not lead to a meaningful alteration in the capacitance as confirmed at the end of the experiment (Fig. 11*B*) and throughout its duration (Fig. 11*C*). Likewise, Figure 11, *D*–*F* illustrate that inhibition of MPC with MSDC-0160 also did not produce significant changes in the capacitance at either concentration. We then analyzed changes in the resistance of HRECs following the inhibition of MCT1 and MPC. Results shown in Figure 12*A* indicate that resistance did not significantly differ from the control group when treated with AZD3965 (1 and 10 μM); these results were consistent at the endpoint of the experiment (Fig. 12*B*) and throughout the experimental period (Fig. 12*C*). Similarly, as illustrated in Figure 12*D*, treatment with MSDC-0160 (1 and 10 μM) did not lead to significant changes in the resistance of HRECs, as observed both at the end of the experiment (Fig. 12*E*) and throughout the experimental period (Fig. 12*F*). Collectively, these findings suggest that neither MCT1 nor MPC plays significant roles in regulating HREC functions.

## Discussion

The key finding of this study is that lower glycolytic components have distinct effects on HREC functionality, with GAPDH and its downstream products (1,3-BPG and NADH), playing a critical role in maintaining endothelial barrier integrity and promoting cell spreading, two processes vital in retinal diseases, including diabetic retinopathy. This conclusion is supported by multiple lines of evidence from our *in vitro* and *in vivo* experiments. First, pharmacological inhibition of GAPDH with heptelidic acid caused a dose-dependent decrease in electrical resistance and an increase in capacitance in HRECs, without inducing cell death, indicating that GAPDH activity is crucial for HREC function. Second, siRNA-mediated GAPDH knockdown led to a significant reduction in endothelial resistance, mirroring the effects observed with heptelidic acid, confirming that the barrier-disrupting effects were directly linked to GAPDH inhibition rather than off-target effects of the drug. Furthermore, *in vivo* FA demonstrated that intravitreal injection of heptelidic acid resulted in retinal vascular leakage, accompanied by a significant increase in albumin extravasation, indicating that GAPDH inhibition disrupts endothelial integrity in both cellular and animal models. Lastly, inhibition of GAPDH downstream glycolytic enzymes that accumulate 1,3-BPG and NADH, such as PGK with NG-52 or PKM with shikonin, enhanced barrier resistance and promoted cell spreading in HRECs. In contrast, blocking other lower glycolytic components, including LDH, MCT, and the mitochondrial pyruvate carrier, did not significantly affect HREC barrier function or cell spreading, highlighting a specific role for GAPDH in endothelial integrity. To the best of our knowledge, this study is the first to reveal the relationships between HREC functions in response to inhibitors targeting various lower glycolytic components, integrating ECIS-based mathematical modeling with both genetic and pharmacological approaches to elucidate the role of GAPDH in regulating endothelial barrier integrity and vascular stability.

The role of GAPDH in regulating cell functionality is controversial due to its involvement in both metabolic (29) and nonmetabolic processes (30). For example, studies on cultured cerebellar neurons have shown that increased GAPDH levels and its translocation to the nucleus are associated with neuronal death, triggered by either aging (31, 32) or cytosine arabinonucleoside exposure (33). In these cases, antisense oligonucleotides targeting GAPDH mRNA or protein synthesis inhibitors prevented GAPDH's nuclear translocation and provided a protective effect (31, 33). Similarly, GAPDH has been implicated in DR in diabetic rats, where its nuclear translocation, along with ADP ribosylation and nitration, contributed to cell death in retinal vascular endothelial cells, leading to the formation of acellular capillaries (34). High glucose levels also prompted GAPDH's nuclear translocation, resulting in apoptosis in retinal Müller cells (35). While these studies highlight GAPDH's pro-apoptotic role, others point to its protective function, particularly in cancer progression. GAPDH is often overexpressed in human cancers (36), where it helps tumor survival by coordinating glycolysis (to produce ATP) and autophagy (to remove damaged mitochondria (37)), thereby protecting cells from caspase-independent cell death (38). In support, it has been reported that high-glucose conditions inhibit cytosolic GAPDH activity, resulting in adherens junction disassembly and endothelial permeability (39). This apparent contradiction may be explained by GAPDH’s dual role: in the cytoplasm, it promotes survival by engaging in glycolysis, while its translocation to the nucleus triggers cell death (40). Despite this complexity, the role of GAPDH in retinal endothelial cells under normal physiological conditions has not been well-studied. Our study is the first to demonstrate GAPDH's critical role in maintaining barrier integrity and cell spreading in HRECs under nonpathological conditions. Specifically, we found that inhibiting the glycolytic function of GAPDH with heptelidic acid did not induce cell death, but it irreversibly disrupted essential cellular activities, including the cell spreading and integrity of the endothelial barrier. These findings shed light on GAPDH's multifaceted role in endothelial cell biology and its implications for disease.

Heptelidic acid, also known as koningic acid, inhibits GAPDH by binding to its active site and interfering with its enzymatic function in glycolysis. GAPDH plays a critical role in the glycolytic pathway, catalyzing the conversion of glyceraldehyde-3-phosphate into 1,3-BPG with the concurrent reduction of NAD^+^ to NADH. Heptelidic acid acts as a selective, irreversible inhibitor by forming a covalent bond with the cysteine residue in the active site of GAPDH. This covalent modification prevents GAPDH from binding its natural substrate (glyceraldehyde-3-phosphate) and carrying out the catalytic reaction (41). As a result, the glycolytic pathway is interrupted, leading to a reduction in the production of 1,3-BPG and a disruption of NADH generation from glycolysis. The specificity of heptelidic acid for GAPDH makes it a valuable tool for studying the effects of glycolytic inhibition in various cellular processes (11), including endothelial barrier function and cellular metabolism in normal and disease conditions.

One key mechanism contributing to endothelial dysfunction upon GAPDH inhibition by heptelidic acid is the decrease in NADH production (Fig. 5*C*). NADH is crucial for maintaining redox balance and ATP production through mitochondrial oxidative phosphorylation, specifically at complex I, which yields approximately three molecules of ATP [reviewed in (42)]. The decrease in NADH levels impairs complex I activity, resulting in diminished energy reserves essential for supporting the endothelial barrier and cell spreading. Our previous findings have established that complex I is the most important component in regulating the barrier integrity, cell adhesion, and spreading of HRECs (26). Another mechanism contributing to endothelial dysfunction upon GAPDH inhibition by heptelidic acid is the reduction of 1,3-BPG, a critical intermediate in glycolysis involved in actin polymerization and cell motility (43). The decrease in 1,3-BPG disrupts actin filament formation, compromising cytoskeletal stability, cell shape, and the integrity of intercellular junctions. As a result, the endothelial barrier becomes more permeable, with impaired cell adhesion, further contributing to endothelial dysfunction. Lastly, GAPDH has been recognized as a critical regulator in the transition from upper to lower glycolysis (7), and its inhibition disrupts downstream glycolytic processes required for ATP production. ATP is crucial for maintaining cytoskeletal dynamics, cell adhesion, and tight junctions, all of which are essential for preserving endothelial barrier integrity.

It's important to note that while PGK and PKM are critical in regulating lower glycolysis and generating two ATP molecules through substrate-level phosphorylation, inhibiting PGK with NG52 and PKM with shikonin surprisingly led to improved barrier function and enhanced cell spreading in HRECs. This was evidenced by increased resistance (Fig. 9) and decreased capacitance (Fig. 8), suggesting a more stable barrier and enhanced cellular cohesion. This intriguing finding indicates that the accumulation of upstream glycolytic intermediates, such as NADH and 1,3-BPG, may have a protective effect on endothelial barrier function. In particular, NADH fuels complex I activity, which is crucial for supporting the endothelial barrier and cell spreading, resulting in the generation of three ATP molecules. Additionally, HRECs may activate alternative pathways [reviewed in (44)] to bypass the inhibition of PGK and PKM. These pathways include the alanine transaminase pathway, where alanine is converted into pyruvate, and the action of malic enzyme, which converts malate from the Krebs cycle or cytosol into pyruvate. The glutaminolysis pathway also aids in pyruvate generation by converting glutamine into alpha-ketoglutarate, which feeds into the Krebs cycle to produce malate and, subsequently, pyruvate. Another mechanism, lactate recycling, involves lactate being converted back into pyruvate by LDH. These alternative pathways provide metabolic flexibility, ensuring sufficient pyruvate production even when the classical glycolytic route is inhibited.

It is worth mentioning that while glycolysis is crucial for HREC functions, specific enzymes within the pathway play distinct roles, and not all glycolytic steps equally influence HREC behavior. For example, inhibiting lower glycolytic components such as LDH, MCT, and the MPC did not significantly affect HREC barrier integrity or cell spreading. This contrasts with findings in liver and kidney endothelial cells, where lactate promotes vascular permeability by inducing VE-cadherin cleavage and endocytosis through the activation of the GPR91 receptor (45). The discrepancy may be due to differences in GPR91 expression; liver and kidney endothelial cells rely on GPR91 for lactate signaling and increased permeability (45), whereas HRECs express lower levels of GPR91 (46). In patients with diabetes mellitus, increased LDH levels have been linked to the progression of diabetic retinopathy (47), though it remains unclear if LDH elevation is a causative factor or merely a byproduct of the disease. Furthermore, while downregulation of MPC in tumor endothelial cells is linked to angiogenesis and tumor progression (21), the lack of response in HRECs to MPC disruption suggests that these cells behave differently than tumor cells.

In conclusion, this study sheds light on the distinct roles of lower glycolytic components in regulating HREC functionality. It underscores the critical importance of GAPDH and its downstream products (NADH and 1,3-BPG) in maintaining barrier integrity and supporting HREC adhesion and spreading. These findings provide a foundation for developing targeted therapeutic strategies aimed at modulating HREC bioenergetics to address endothelial dysfunction in retinal disorders, while minimizing potential adverse effects on healthy endothelial cells.

## Experimental procedures

### Animal preparation and experimental design

All animal procedures were approved by the Institutional Animal Care and Use Committee of Wayne State University. WT C57BL/6J mice (The Jackson Laboratory) were housed in the Division of Laboratory Animal Resources at Wayne State University in clear plastic cages under standard conditions, including a 12 h light/dark cycle and a controlled temperature of 22 to 24 °C. Mice had unrestricted access to food and water, with light intensity maintained at 1.5-foot candles to minimize the risk of light-induced retinal damage. Intravitreal injections were performed using a Hamilton syringe fitted with a beveled 33-gauge microneedle, as previously described (48). Briefly, mice were anesthetized using 2% isoflurane in oxygen, followed by the application of topical anesthesia (proparacaine HCl, Alcon) and a pupil dilator (tropicamide 1%, Alcon) before injection. One group received a 2 μl intravitreal injection of heptelidic acid (dissolved in DMSO and diluted with PBS to achieve a final concentration of 10 μM in the vitreous volume), while control mice received an equivalent injection of vehicle (DMSO diluted with PBS). One week after intravitreal injections, mice underwent fluorescein angiography to evaluate retinal vasculature and permeability *in vivo*, which was further confirmed by assessing albumin leakage following sacrifice and retinal extraction.

### FA experiment

Mice were anesthetized using 2% isoflurane in oxygen, and pupillary dilation was achieved with 1% tropicamide eye drops. Mice were then positioned on the imaging platform of the Phoenix Micron IV retinal imaging microscope (Phoenix Research Laboratories). To maintain corneal hydration, Genteal gel (Alcon) was applied throughout the process. Then, mice received an intraperitoneal injection of 50 μl of 10% fluorescein sodium, followed by the rapid acquisition of fluorescent images over approximately 5 min. Fluorescein leakage, indicative of vascular permeability, was identified by the blurring of vascular margins and the development of diffuse fluorescence. Quantitative analysis of fluorescein leakage between experimental groups was performed by measuring fluorescence intensities using ImageJ software (National Institutes of Health).

### ECIS experiment

We investigated the effects of two concentrations (1 μM and 10 μM) of six different inhibitors targeting lower glycolytic components on retinal endothelial cell functionality using ECISZθ technology (Applied Biophysics Inc.), as previously described (26, 28). The six inhibitors included heptelidic acid (a GAPDH inhibitor) (11), NG52 (a PGK inhibitor) (14), shikonin (a PKM inhibitor) (19), galloflavin (an LDH inhibitor) (49), AZD3965 (an MCT1 inhibitor) (50), and MSDC-0160 (an MPC inhibitor) (51). The experiment started with treating a 96-well array (96W20idf PET; Applied Biophysics Inc.) with 100 μM cysteine (50 μl/well; Applied Biophysics) for 30 min, followed by aspiration. The array was then coated with 0.02% gelatin (50 μl/well; Sigma) for 30 min before being aspirated again. Next, HRECs from Cell Systems were seeded in Microvascular Endothelial Cell Growth Medium-2 BulletKit (Lonza; Catalog #: CC-3202 EGM-2 MV). Once the HRECs reached confluence and formed a mature monolayer (capacitance < 20 nF), the culture media were replaced with media devoid of growth factors for 10 to 12 h prior to applying the various concentrations of inhibitors. We then assessed the resistance and the capacitance of the HREC monolayer over time and frequency by applying a 1 μA alternating current to the electrode array at the base of the 96-well plate, using nine frequencies ranging from 250 to 64,000 Hz. To calculate the normalized resistance or capacitance value at each time point, the corresponding raw value was divided by the value obtained prior to treatment with the glycolytic inhibitors. These data points were collected either at the end of the experiment or throughout the duration by calculating the AUC.

### Assessment of HREC viability

The effect of different concentrations of lower glycolytic inhibitors on HREC viability was evaluated using the LDH Cytotoxicity Assay (CyQUANT; Invitrogen-C20300) at 24 and 48 h. At each time point, 25 μl of supernatant was collected from each ECIS well and transferred to a 96-well plate. An equal volume (25 μl) of LDH detection reagent, prepared according to the manufacturer’s instructions, was added to each sample. The mixture was incubated at room temperature for 30 min and protected from light. Absorbance was measured at 490 nm and 680 nm using a Synergy H1 Hybrid Multi-Mode Microplate Reader (BioTek Instruments), with the 680 nm reading subtracted to account for background noise. Cytotoxicity was quantified by normalizing the corrected 490 nm absorbance reading to the control, and results were expressed as a percentage of LDH release relative to the control group.

### The rate of NADH formation

The rate of NADH formation over ∼50 h was measured using the NADH-Glo Assay Kit (Promega, Cat. #G9071). Briefly, HREC lysates were prepared by lysing cells in a base solution containing dodecyltrimethylammonium bromide, which disrupts cells while preserving dinucleotide stability. To selectively measure NADH, sample lysates were heated at 60 °C for 15 min to degrade NAD^+^. The collected samples were then neutralized by adding 50 μl of HCl/Trizma solution. A 50 μl aliquot of each sample was mixed with 50 μl of NADH-Glo Detection Reagent in a 96-well plate, incubated at room temperature for 30 min, and luminescence was measured using a Varioskan LUX plate reader (Thermo Fisher Scientific).

### Silencing of GAPDH with siRNA

As described previously (9, 52), HRECs at 80% confluency were used for the assay. GAPDH siRNA (hs.Ri.GAPDH.13.2, Integrated DNA Technologies) and Lipofectamine 2000 transfection reagent (Cat. #11668019, Life Technologies, Thermo Fisher Scientific) were separately diluted in Opti-MEM medium (Cat. #11058021, Gibco, Thermo Fisher Scientific), mixed, and incubated for 5 min at room temperature. The transfection mixture was then added to HRECs seeded on ECIS plates and incubated for 24 h. Cells treated with control Dicer-substrate RNA (DsiRNA) duplexes (Integrated DNA Technologies) served as a negative control (scramble siRNA). After 24 h, the transfection medium was replaced with a fresh medium that did not contain the transfection reagent, and cells were maintained under this condition until the experiment ended. At the endpoint, cells were lysed and subjected to Western blot analysis to assess GAPDH knockdown efficiency.

### Western blotting analysis

Mouse retinae and HRECs were lysed in RIPA buffer supplemented with protease and phosphatase inhibitors, following established protocols (53). To assess GAPDH protein expression in HREC lysates, the samples were incubated with an anti-GAPDH antibody (Cell Signaling Technology, Cat. # 2118). To assess albumin leakage in the retina, retinal tissue lysates were incubated with an HRP-conjugated anti-albumin antibody (Bethy, Fortis Life Sciences, Cat. # A90-134P). The internal control was β-actin, detected using an anti-β-actin antibody (Santa Cruz Biotechnology, Cat. # sc-47778). After primary antibody incubation, detection was performed with the corresponding HRP-conjugated secondary antibody and visualized using an enhanced chemiluminescence detection system (Thermo Fisher Scientific). Protein bands were imaged with the iBright Imaging System (Thermo Fisher Scientific), and densitometric analysis was performed using ImageJ software.

### Statistical analysis

Statistical comparisons among the experimental groups were performed using one-way ANOVA. To address multiple comparisons, the false discovery rate was controlled using the two-stage linear step-up procedure of Benjamin, Krieger, and Yekutieli, with a threshold of <0.05. For comparisons between two groups, an unpaired *t* test was used. The *p*-values were visually depicted as follows: ∗ for *p* ≤ 0.05, ∗∗ for *p* ≤ 0.01, ∗∗∗ for *p* ≤ 0.001, and ∗∗∗∗ for *p* ≤ 0.0001. The figure legends included the number of biological replicates for each analysis.

## Data availability

Additional data available upon request of the corresponding author.

## Conflict of interest

The authors declare that they have no conflicts of interest with the contents of this article.
